# Establishment and assessment of an oral squamous cell carcinoma N7-methylguanosine methyltransferase associated microRNA prognostic model

**DOI:** 10.7150/jca.98350

**Published:** 2024-09-30

**Authors:** Jianrong Li, Chu Li, Xiaolian Li, Yuling Chen, Zhangfu Li, Yuntao Lin, Huan Jing, Yufan Wang, Hongyu Yang

**Affiliations:** 1 School of Stomatology, Zunyi Medical University, Zunyi, Guizhou 563000, China.; 2 Department of Oral and Maxillofacial Surgery, Peking University Shenzhen Hospital, Shenzhen, Guangdong 518036, China.

**Keywords:** oral squamous cell carcinoma, N7-methylguanosine methyltransferase, microRNA, prognosis, immune microenvironment

## Abstract

**Background:** N7-methylguanosine (m7G) methyltransferases and microRNAs (miRNAs) are closely associated with tumor progression. However, the role of m7G methyltransferase-related miRNAs as prognostic markers in oral squamous cell carcinoma (OSCC) has not been studied. This study aimed to explore the m7G methyltransferase-related miRNAs in OSCC, establish a prognostic model based on m7G methyltransferase-related miRNAs, investigate their correlation with immune cell infiltration, and assess their potential prognostic value.

**Methods:** Transcriptional and clinical data of patients with OSCC were obtained from The Cancer Genome Atlas (TCGA) database. TargetScan and miRWalk were used to predict m7G methyltransferase-related miRNAs. Subsequently, differentially expressed m7G methyltransferase-related miRNAs in TCGA-OSCC were selected. Cox and least absolute shrinkage and selection operator (LASSO) regression analyses were used to build an m7G methyltransferase-related miRNA risk prognostic model for TCGA-OSCC. Patients were stratified into high- and low-risk groups. The predictive and diagnostic accuracies of the risk prognostic model were further validated using Kaplan-Meier survival analysis, receiver operating characteristic (ROC) curve analysis, independent prognosis analysis, and nomogram plots. Finally, quantitative real-time polymerase chain reaction (qPCR) was used to validate the expression levels of m7G methyltransferase-related miRNAs in postoperative cancer and adjacent normal tissues from 60 patients with OSCC.

**Results:** Through Cox and LASSO regression analysis, six candidate miRNAs (hsa-miR-338-3p, hsa-miR-1251-3p, hsa-miR-3129-5p, hsa-miR-4633-3p, hsa-miR-216a-3p, and hsa-miR-6503-3p) most relevant to the prognosis of patients with OSCC were identified to construct an m7G methyltransferase-related miRNA risk prognostic model. In this model, the overall survival (OS) of the high-risk group was significantly shorter than that of the low-risk group (P < 0.001). The model effectively predicted prognosis and served as an independent prognostic indicator for patients with OSCC. Compared with the low-risk group, the high-risk group exhibited a significantly increased capacity for immune cell infiltration (P < 0.05), while the activation and initiation abilities of immune cells were decreased. Finally, six m7G methyltransferase-related miRNAs were validated in OSCC tissue samples.

**Conclusion:** The risk prognostic model based on six m7G methyltransferase-related miRNAs can predict the OS rate of patients with OSCC and has the potential to guide individualized treatment. This prognostic model is closely associated with immune cell infiltration in patients with OSCC.

## 1. Introduction

Head and neck squamous cell carcinoma (HNSCC) is the sixth most common cancer, with approximately 700,000 diagnosed cases 1,2. Oral squamous cell carcinoma (OSCC) accounts for approximately 90% of all malignant tumors of the head and neck region 1,3. OSCC is a heterogeneous tumor originating from the inner layer of the oral mucosa, with a global incidence, particularly in developing countries. Its incidence is higher in male patients than in female patients 4. Although primary tumor resection remains the standard treatment 5, OSCC's high invasiveness often leads to postoperative recurrence and metastasis 6, resulting in a relatively low survival rate of 5 years 7. Although targeted therapy and immunotherapy have improved outcomes 8,9, their application is limited by individual variability and issues related to drug resistance 10. Therefore, effective biomarkers are needed to predict OSCC prognosis, enable accurate assessment of patient prognosis, and guide subsequent treatment.

N7-methylguanosine is a commonly occurring RNA post-transcriptional modification formed by methylation of the seventh nitrogen atom of guanine at the RNA purine position 11. It plays vital roles in different RNA stages such as RNA transcription, processing, degradation, and translation. In addition to being presented at 5' end and internal positions of mRNA in eukaryotes 12-14, m7G modification is also widely found in rRNA, tRNA, and miRNA 15,16. However, unusual m7G modifications are often linked to tumor occurrence and progression 17. The m7G regulatory factors methyltransferase 1 (METTL1) and WD Repeat Domain 4 (WDR4) participate in the regulation of various cancer types, including HNSCC, liver cancer, bladder cancer, and lung cancer, by changing the m7G modification levels of miRNAs and tRNAs 18,19. METTL1 and WDR4 are significantly upregulated, increasing m7G modifications in tRNAs, which enhances oncogenic mRNA translation, promoting tumor progression and poor prognosis. METTL1 knockout alters the link between cancer cells and their microenvironment in HNSCC. The ratio of CD4^+^ T cells to Tregs is significantly reduced, whereas the permeation of CD4^+^ memory T cells, CD4^+^ naïve T cells, and CD8^+^ naïve T cells increases 19. These findings highlight the crucial roles of METTL1 and WDR4 in HNSCC.

MicroRNAs (miRNAs) are single-stranded noncoding RNAs with lengths ranging from 19-25 nucleotides. They have vital functions in post-transcriptional regulation by binding to mRNAs and long noncoding RNAs that influence important biological processes such as cell proliferation, differentiation, and apoptosis 20. In addition, miRNAs are closely related to immune response and angiogenesis 21,22. However, studies have confirmed that the dysregulation of miRNAs is closely linked to cell metastasis and drug resistance in cancer cells. Several miRNAs have been identified as possible diagnostic and prognostic biomarkers for OSCC and used to construct prognostic models 23-26. However, the role of m7G methyltransferase-related miRNAs in OSCC remains unclear. Therefore, it is essential to further investigate their significance as prognostic biomarkers for patients with OSCC and provide insights into novel OSCC treatment methods.

Based on this evaluation, we predicted the upstream miRNAs associated with the N7-methyltransferase METTL1 and the WDR4 complex. Using the TCGA database, a risk prognosis model for m7G methyltransferase-related miRNAs was constructed and validated in our study.

## 2. Materials and Methods

### 2.1 Data Collection and Processing

Transcriptome mRNA sequencing data (240 tumor and 17 normal samples) and miRNA sequencing data (245 tumor and 17 normal samples) of patients with OSCC were acquired from the TCGA database (https://portal.gdc.cancer.gov/) 27. Corresponding clinical information, including patient age, sex, tumor grade, TNM stage, survival time, and tumor status, was also retrieved. We processed the data using Perl software by parsing and extracting mRNA and miRNA expression levels, generating respective expression matrices with genes or miRNAs as rows and samples as columns. We created a clinical data matrix with patients in rows and clinical features in columns. These matrices were integrated based on patient identifiers to correlate all the data types for each patient. The quality control steps included checking for missing values, normalizing expression data, and filtering out low-quality features, ensuring that subsequent analyses were based on high-quality and well-integrated datasets 27.

### 2.2 m7G Modification-Related miRNA Screening and Enrichment Analysis

The R package "limma" was employed for comparison of expression intensities of both m7G methyltransferase genes *METTL1* and *WDR4* in TCGA-OSCC tissues and normal tissues. Next, upstream miRNAs targeting *METTL1* and *WDR4* were predicted using the TargetScan database (https://www.targetscan.org/) and miRWalk (http://mirwalk.umm.uni-heidelberg.de/), respectively. This intersection was used to obtain a list of miRNAs related to m7G modifications. Subsequently, the "edgeR" R package was utilized to analyze the expression intensities of these m7G modification-associated miRNAs inside TCGA-OSCC and normal tissues. FDR < 0.05 and |log_2_Foldchange| > 1 were applied to identify significantly differentially expressed m7G modification-related miRNAs, and a network relationship diagram was drawn using the Cytoscape 2.8 tool 28. Finally, the FunRich tool was used to enhance the differential expression of these m7G modification-associated miRNAs.

### 2.3 Construction and Evaluation of Prognostic Model with Basis on m7G Modification-Related miRNAs

Initially, 245 patients with OSCC were randomly categorized into training and testing sets in a ratio of 7:3. The training set comprised 172 patients for model construction and the testing set included 73 patients to validate the model's performance. Subsequently, the Kaplan-Meier "survival" R package was used for Cox regression analysis to assess the prognostic significance of the differentially expressed m7G modification-associated miRNAs (P < 0.05). The "glmnet" R package was then applied for least absolute shrinkage and selection operator (LASSO) to refine and select miRNAs for constructing the prognostic model. The risk score calculation formula for each patient was described as follows: Risk Score = (0.326 * expression of hsa-miR-338-3p) + (1.597 * expression of hsa-miR-1251-3p) + (2.288 * expression of hsa-miR-4633-3p) + (0.743 * expression of hsa-miR-216a-3p) + (0.348 * expression of hsa-miR-6503-3p) - (0.494 * expression of hsa-miR-3129-5p).

Next, using the median risk scores in the training set as the threshold, patients with OSCC in the training, testing, and entire TCGA sample sets were stratified into high- or low-risk groups for subsequent examination and validation. The Kaplan-Meier curve (log-rank analysis) was employed to assess the survival level differences between the high- and low-risk groups. A prime component evaluation was utilized to evaluate accurate grouping, and model accuracy was assessed by calculating the area under the receiver operating characteristic (ROC) curve. A concordance index was used to assess the discriminative ability of the model. Finally, univariate and multivariate Cox proportional hazards analyses were conducted to evaluate the risk scores of the model to check the clinical features and self-regulating predictive biomarkers in patients with OSCC.

### 2.4 Nomogram Construction

To enhance clinical applicability, the "rms" R package was used to create a nomogram, integrating model risk scores and patient clinical data. This nomogram helped quantify factors affecting the analysis of patients with OSCC and predict the survival period of patients. A calibration curve was then generated to assess the model's predictive accuracy, demonstrating consistency between predicted and actual survival times.

### 2.5 Association Analysis between Prognostic Model and Tumor Immune Microenvironment

The R packages "estimate,” "GSEABase," and "GSVA" were employed to execute the ESTIMATED algorithm as well as conduct single sample Gene Set Enrichment Analysis 29. This allowed the evaluation of 23 immune cell infiltrations and 13 immune-associated functional scores for each TCGA-OSCC model. Subsequently, we assessed immune cell penetration and patient immunity within the high- and low-risk groups. In addition, expression level difference analyses were performed to identify checkpoints for immune-associated genes among the various subgroups. Finally, to evaluate the antitumor immune function scores for each patient, we used Immunophenotype Element Server and Tracking Tumor (http://biocc.hrbmu.edu.cn/TIP/index.jsp) to compare the antitumor immunity systems between the two risk groups.

### 2.6 Prediction of Potential Regulatory Mechanisms and Sensitivity for Potential Therapeutic Drugs between High- and Low-Risk Subgroups

Enhancement of gene sets was employed to identify signal passages and differentially expressed genes between the high- and low-risk groups. This was performed to unveil the potential regulatory mechanisms contributing to the prognostic differences between these two risk subgroups. Additionally, utilizing R software and the "pRRophetic" package, we calculated half- maximal inhibitory concentration (IC_50_), commonly used to assess antitumor drug efficacy. Drug sensitivity was also detected in the high- and low-risk groups. Lower IC_50_ values indicated high sensitivity of the drugs for treatment.

### 2.7 Detection of Relative m7G Expression Levels in Modification-Related miRNAs within Clinical OSCC Tissues

Clinical OSCC tissues and paired adjacent normal tissues were acquired from 60 patients at the Oral and Maxillofacial Surgery Section of the Peking University Shenzhen Hospital. These patients had not undergone cancer treatment before surgery. The tissue samples were rapidly transferred and stored in liquid nitrogen, which was approved by the Ethical Society of Peking University Shenzhen Hospital (grant number: 2022-117). Informed consent was obtained from all patients or their relatives. Total RNAs was purified from OSCC and normal tissues using TRIzol reagent (Takara Bio Inc., Kusatsu, Japan). cDNA for miRNA examination was generated using the stem-loop method (Accurate Bio Inc., Hunan, China) 13. Subsequently, real-time quantitative PCR was performed using SYBR^®^ Green Premix *Pro Taq* HS qPCR Kit II (Accurate Bio Inc, Hunan, China) on a LightCycler® 480 PCR instrument (Roche, Indianapolis, IN, USA). The expression of the six m7G methyltransferase-associated miRNAs were normalized to U6 and calculated using the 2^^-ΔΔCt^ method in triplicate. The primer sequences are listed in Supplementary Table 1.

### 2.8 Statistical Analysis

The statistical analysis of public data was performed using R (version 4.2.1), and experimental data were visualized and statistically analyzed using GraphPad Prism 9.5.1. Significance testing for differences was conducted using the Wilcoxon test, and correlation analysis was performed using the Spearman's rank relationship method, Statistical significance was set at p<0.05.

## 3. Results

### 3.1 Identification of Differentially Expressed m7G Methyltransferase-Associated miRNAs in TCGA-OSCC Tissues

**Figure 1:**
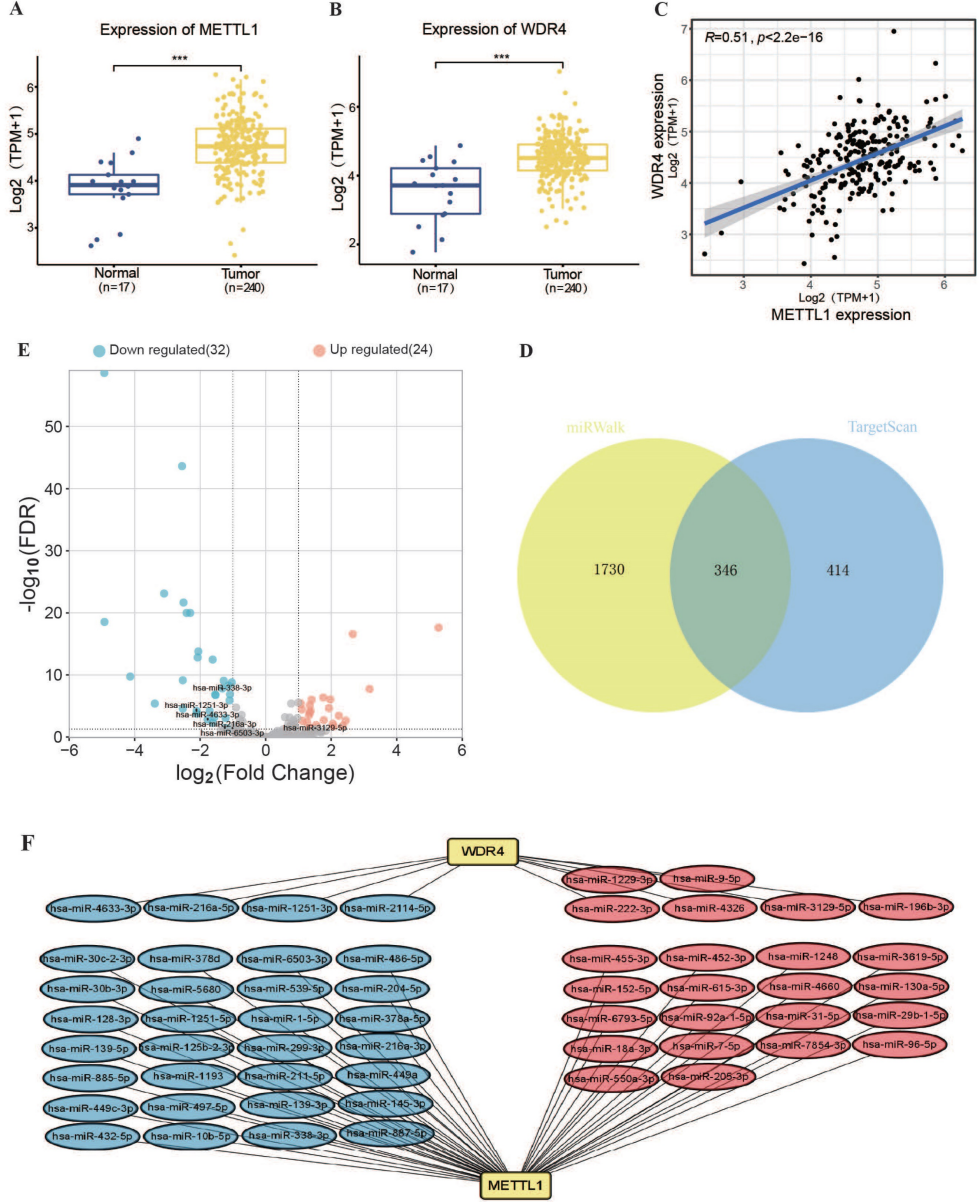
** Expression and regulatory network analysis of m7G methyltransferase complex *METTL1*/*WDR4* mRNA and associated miRNAs in TCGA-OSCC. (A, B)** *METTL1* and *WDR4* expression levels in the TCGA-OSCC cohort. **(C)** Correlation evaluation of expression between *WDR4* and *METTL1*. **(D)** Prediction scores of miRNAs binding to *METTL1* and *WDR4* mRNA using TargetScan and miRWalk online databases (Venn diagram). **(E)** Volcano plot depicting differential expressed m7G methyltransferase-associated miRNAs between normal and tumor tissues in the TCGA-OSCC cohort.** (F)** Co-expression network of miRNA-m7G-associated genes.

Analysis of TCGA transcriptome data revealed that the RNA levels of m7G methyltransferase *METTL1* and *WDR4* were significantly higher in OSCC tissues than in normal tissues (Figure 1A, B). *METTL1* and *WDR4* were significantly and positively correlated (r = 0.51, P < 0.001; Figure 1C). Subsequently, the upstream miRNAs related to the m7G methyltransferase *METTL1-WDR4* complex were predicted using the TargetScan and miRWalk online databases (Figure 1D). In total, 346 miRNAs associated with m7G methylation were identified. Among them, 56 miRNAs were differentially expressed in TCGA-OSCC tissues (Figure 1E), with 24 upregulated and 32 downregulated miRNAs (FDR < 0.05, |log_2_ Fold Change| > 1). Among the 56 miRNAs, 46 targeted *METTL1* and 10 targeted *WDR4* (Figure 1F).

### 3.2 Functional Evaluation for m7G Methyltransferase-Associated miRNAs in TCGA-OSCC

**Figure 2:**
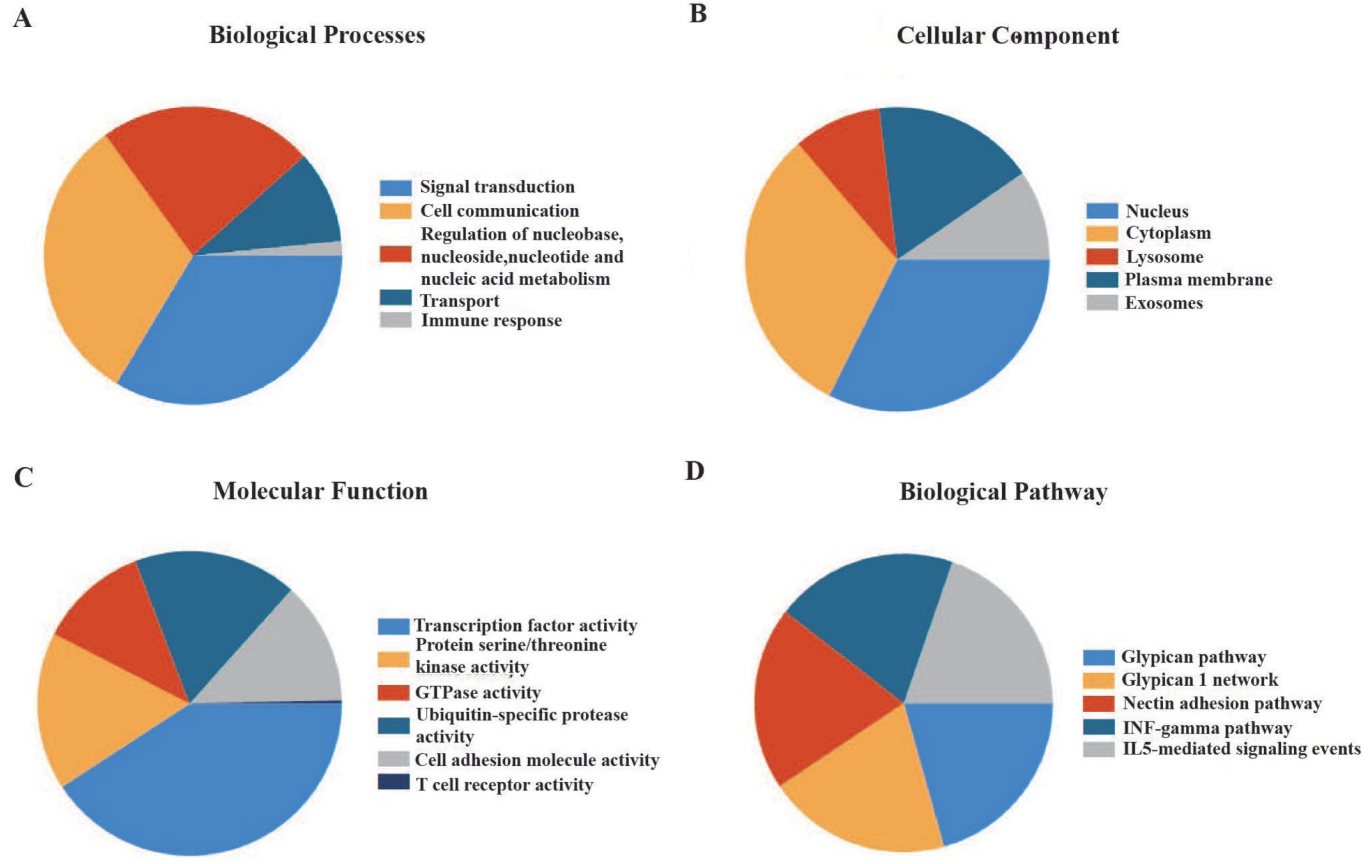
** Enrichment analysis of 56 m7G methyltransferase-associated miRNAs. (A)** Biological processes,** (B)** cellular components, **(C)** molecular functions, and **(D)** biological pathways of m7G methyltransferase-associated miRNAs.

Functional enhancement evaluation of m7G methyltransferase-associated miRNAs using the FunRich tool revealed enrichment in 89 items related to biological processes, 147 items related to molecular functions, 443 items related to cellular components, and 494 items related to biological pathways. In terms of biological processes, these miRNAs were significantly enriched in cell communication, signal transduction, nucleic acid metabolism, and immune response (Figure 2A). In terms of cellular components, these miRNAs were distributed in the nucleus, cytoplasm, exosomes, and plasma membrane (Figure 2B). The molecular functions were primarily associated with transcription factor activity, serine or threonine kinase activity, cell adhesion molecule activity, and T-cell receptor activity (Figure 2C). Additionally, m7G methyltransferase-associated miRNAs were found to be involved in glypican-, IFN-γ-, and IL5-mediated signaling pathways (Figure 2D).

### 3.3 Construction and Validation of m7G Methyltransferase-Associated miRNA Prognostic Model

**Figure 3:**
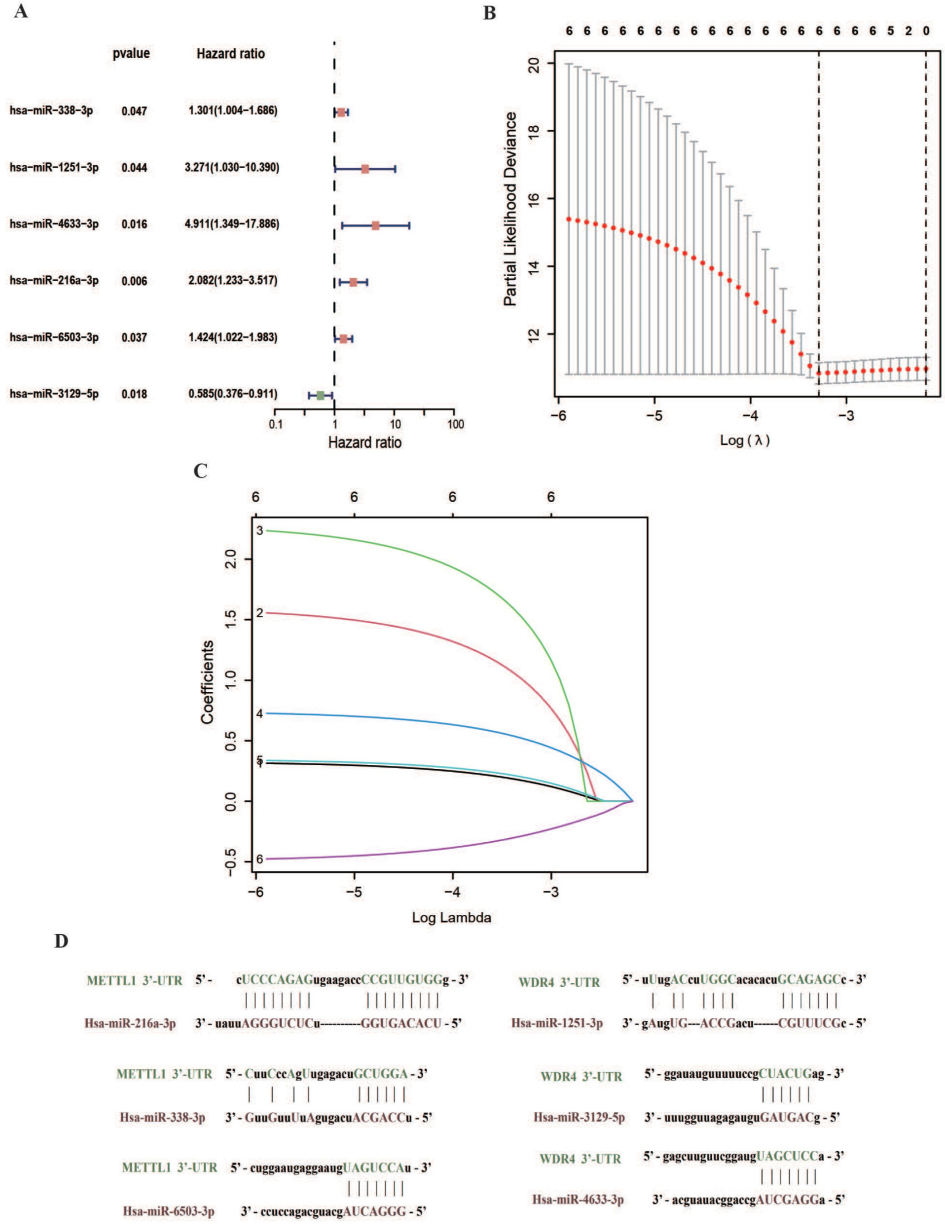
** Establishment of the m7G methyltransferase-associated miRNA prognostic model. (A)** Univariate Cox regression analysis of six m7G methyltransferase-associated miRNAs shown in a forest plot. **(B)** LASSO regression analysis.** (C)** Cross-validation. **(D)** Prediction results of binding sites at the 3'UTR of *METTL1* and *WDR4* mRNA for six miRNAs.

**Figure 4:**
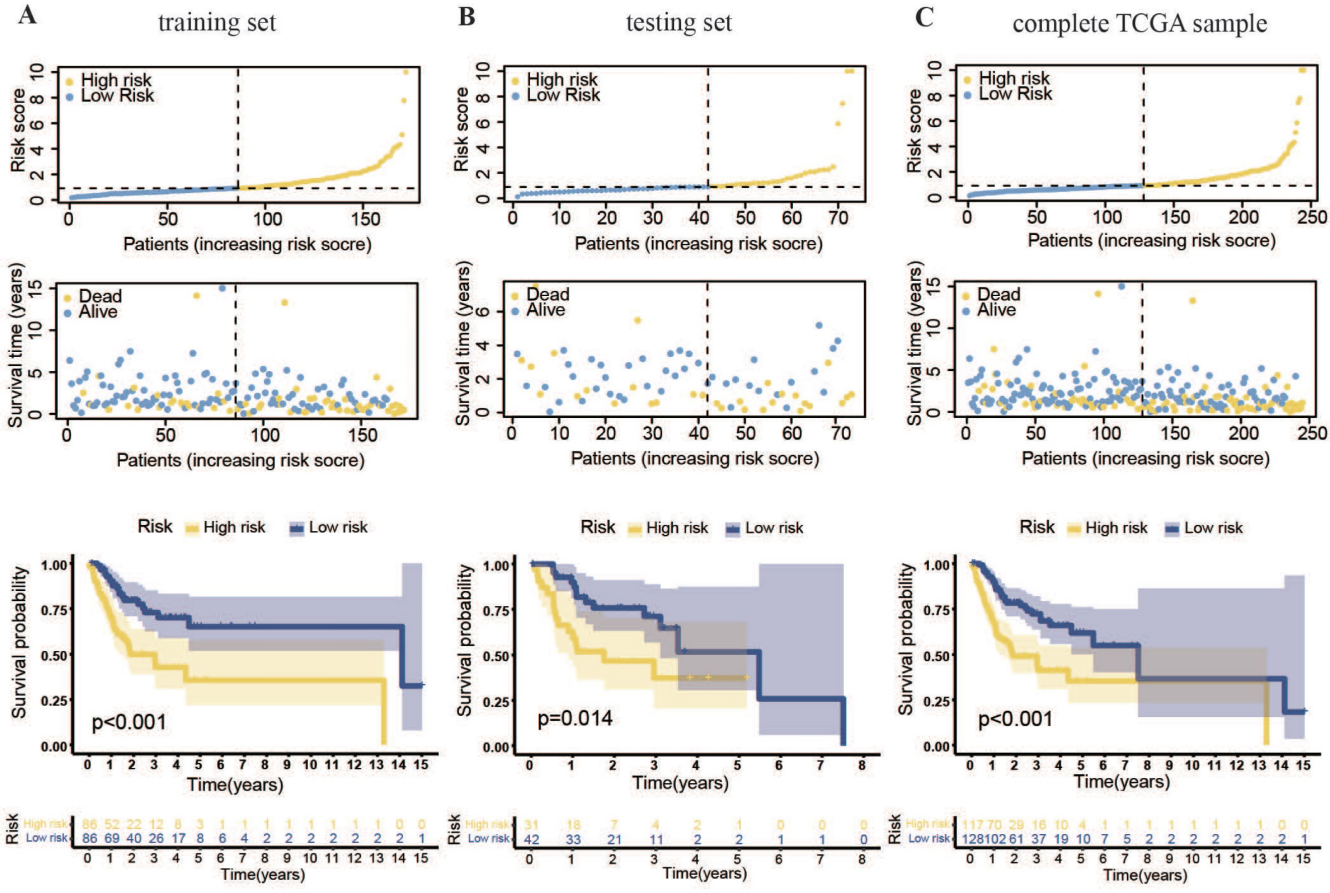
** Prognostic model for m7G methyltransferase-associated miRNAs constructed based on TCGA-OSCC patient data. (A)** Risk value distribution and Kaplan-Meier survival curve for the training set. **(B)** Risk value distribution and Kaplan-Meier survival curve for the testing set. **(C)** Risk value distribution and Kaplan-Meier survival curve for the entire TCGA sample.

Cox and LASSO regression analyses were performed for the 56 m7G methyltransferase-associated miRNAs and six representative prognostic miRNAs (Figure 3A, B). The corresponding Cox coefficients are shown in Table 1. LASSO analysis was validated through cross-validation (Figure 3C). Subsequently, 3'UTR targeting analysis revealed binding sites in the 3'UTR region of the *METTL1* and *WDR4* mRNA for these six miRNAs (Figure 3D). Using these six m7G methyltransferase-associated miRNAs, a predictive risk model was developed for a training set of 172 samples. Based on the median risk score of the training set (0.91896), the 172 TCGA-OSCC cases were categorized into low- or high-risk groups. The number of deaths in the high-risk group was significantly higher than that in the low-risk group (P < 0.001). As the danger score increased, patient deaths also increased (Figure 4A). Subsequently, model applicability was further validated in the test and all-sample sets. Based on the median risk score from the training set, patients in the test and all-sample sets were categorized into low- or high-risk groups (Figure 4B, C). According to the Kaplan-Meier survival curve results, the survival time of the low-risk group was significantly longer than that of the high-risk group.

**Figure 5:**
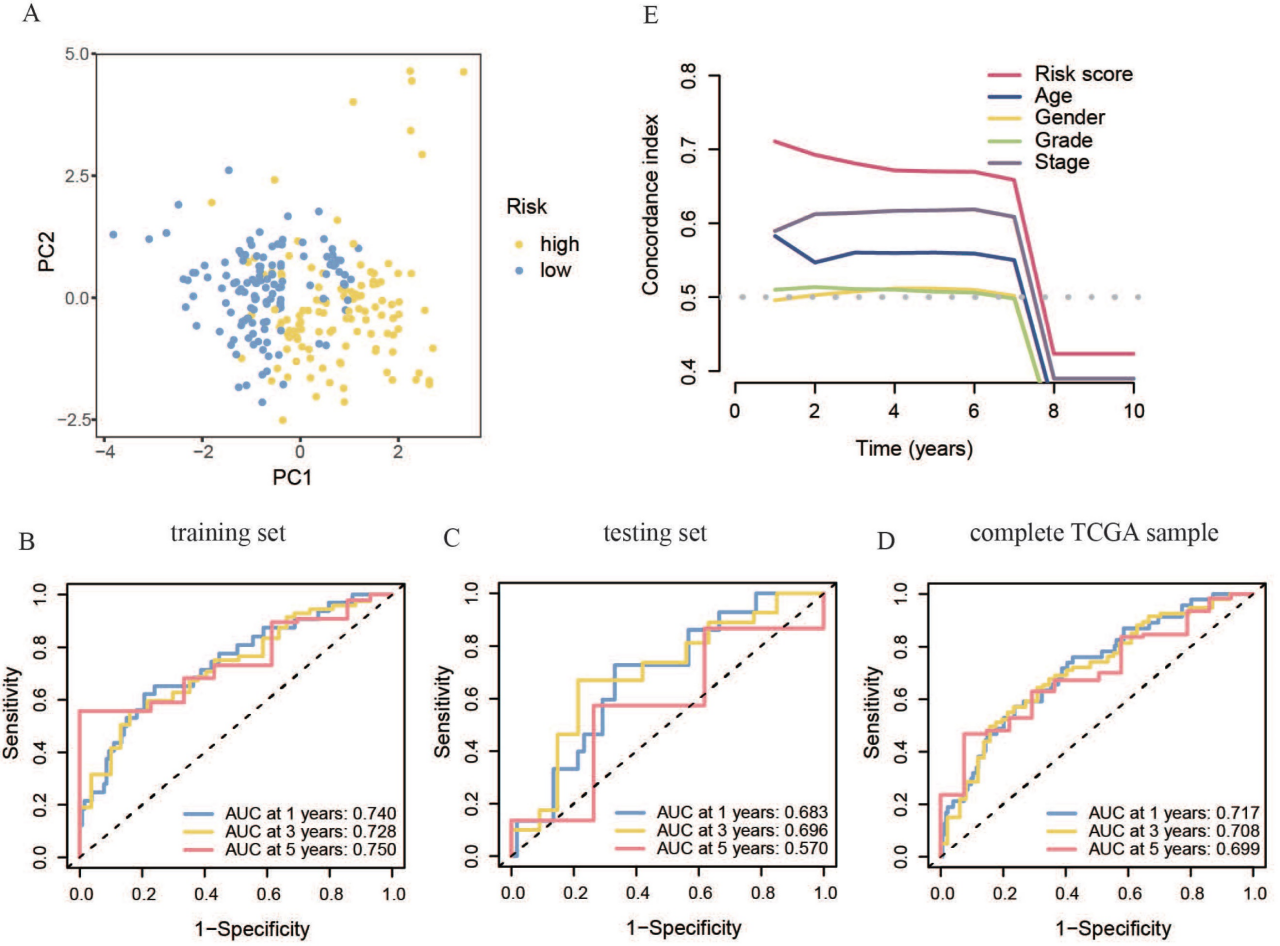
** Accuracy of the constructed m7G methyltransferase-associated miRNA prognostic model in predicting survival rates for patients with OSCC. (A)** Principal component analysis of TCGA whole-sample data. **(B)** Time-reliant ROC curve for the training set. **(C)** Time-reliant ROC curve for the testing set. **(D)** Time-reliant ROC curve for the complete TCGA sample. **(E)** Concordance index analysis comparing risk scores with clinical variables such as age, sex, tumor grade, and TNM stage.

The primer component investigation results for the all-sample set showed that the transcriptional features of the high- and low-risk patient groups were significantly different (Figure 5A). This was characterized by substantial inter-group differences and good intra-group repeatability, indicating distinct transcriptional features between the two risk groups and allowing for effective differentiation. To evaluate the prognostic model, ROC curves were generated for the training set, yielding AUC scores above 0.7 at 1, 3, and 5 years. The model demonstrated strong accuracy in predicting survival at these intervals (Figure 5B). This phenomenon was further confirmed in the test and all-sample sets (Figure 5C, D). Additionally, the concordance index (C-index) of our risk score model was better than that of clinical features, such as age, sex, tumor grade, and TNM phase, confirming its reliability as a diagnostic tool for OSCC (Figure 5E).

### 3.4 Establishment and Validation of Nomogram

**Figure 6:**
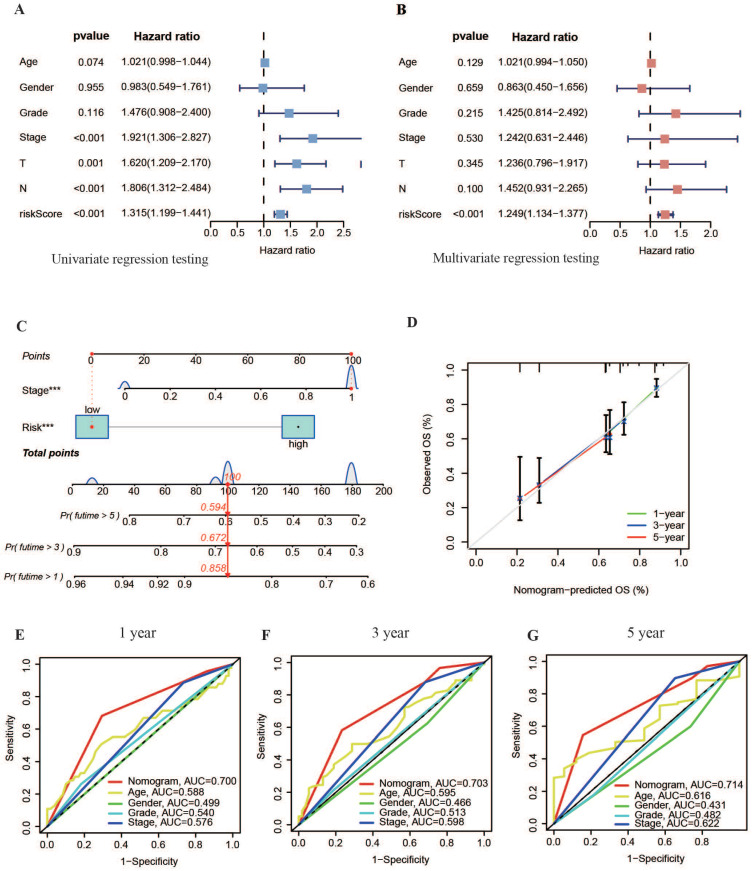
** Construction of a nomogram. (A, B)** Forest plots showing results of univariate and multivariate regression analyses for risk scores. **(C)** Nomogram combining risk values from the prognostic model with patient TNM staging. **(D)** Calibration curve for the nomogram.** (E-G)** ROC curves of the nomogram for predicting survival at 1, 3 and 5 years.

According to the univariate Cox analysis results shown in Figure 6A, the risk score and TNM staging were significantly related to patient diagnosis (P < 0.01). Multivariate Cox regression testing results further indicated that both the risk score and TNM stage could independently serve as prognostic biomarkers for OSCC (P < 0.05), offering valuable insights into patient outcomes (Figure 6B).

Based on the independent prognostic analysis results, a nomogram graph that included the model's risk score and the patient's TNM stage was drawn to increase the application of the experimental model (Figure 6C). This nomogram aimed to determine the overall survival (OS) rate of patients with OSCC at 1, 3 and 5 years. The calibration curve showed increased consistency within the estimated time limit and the actual observed survival time in patients with OSCC (Figure 6D). This result indicated that the survival time of patients with OSCC could be efficiently predicted using the nomogram. Moreover, the nomogram's ROC curves displayed AUC values of approximately 0.7 for 1, 3, and 5 years (s), outperforming other clinical variables (Figure 6E-G). This suggests that the nomogram, along with the basis of the model's danger values, provides more accurate predictions of OS rates in patients with OSCC than predictions based on independent clinical variables. This highlights the nomogram's potential for precise clinical diagnosis.

### 3.5 High- and Low-Risk Groups Exhibited Distinct Tumor Immune Microenvironments

**Figure 7:**
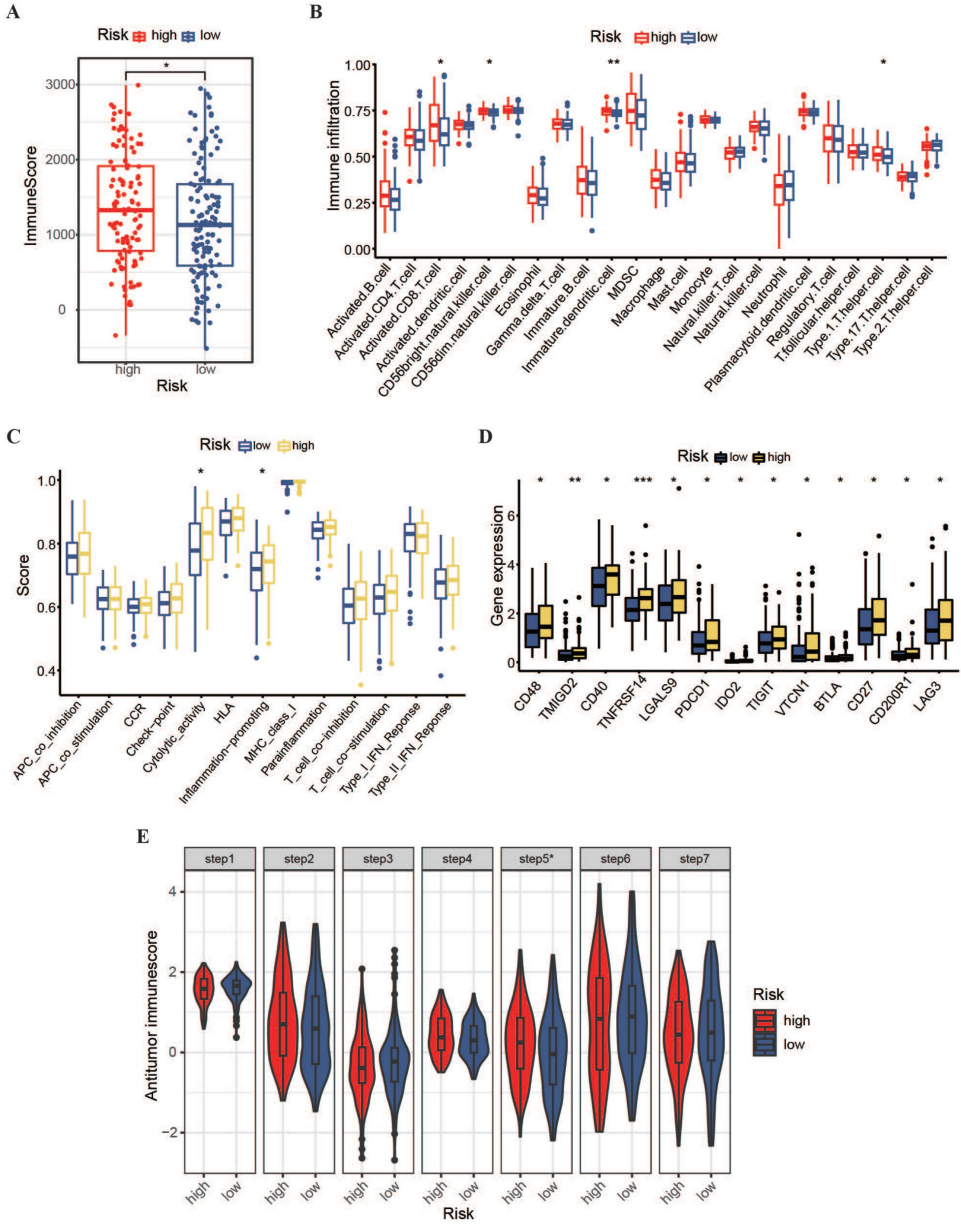
** Immune infiltration and tumor microenvironment in high- and low-risk OSCC groups. (A)** Comparison of immune levels between high- and low-risk groups. **(B)** Comparison of infiltration levels for 23 resistant cell types within high- and low-risk groups. **(C)** Comparison of elevated values for 13 resistance-associated pathways within high- and low-risk groups. **(D)** Comparison of resistant gene expression levels between high- and low-risk groups. **(E)** Assessment of anti-cancer-immune activity in the cancer-immunity cycle for patients with OSCC in the high- and low-risk groups.

To expand the immunotherapy options for patients with OSCC owing to limited treatment options, we conducted immune-related analyses on a prognostic model. Using the ESTIMATE algorithm, we found that the immune score in the high-risk group was higher than that in the low-risk group (Figure 7A), indicating greater immune cell penetration in the high-risk group. This implies a potential link between the risk score and resistant microenvironment in patients with OSCC. Subsequently, the ssGSEA algorithm was used to assess the permeation levels for 23 resistant cell varieties and enrichment scores for 13 protection-related pathways in patients between the two hazardous categories. The results showed that the high-risk group had a stronger potential for resistant permeation and higher proportions of initiated CD8^+^ T cells, type I T helper cells, CD56 natural killer cells, and immature dendritic cells than the low-risk group (Figure 7B). Additionally, cytolytic activity and inflammation promotion were significantly upregulated in the high-risk group (Figure 7C). The expression of multiple immunity-verifying genes was also significantly elevated in the high-risk group (Figure 7D), indicating that the resistant microenvironments of high-risk patients may be abnormally active.

Furthermore, the TIP meta-server method was used to evaluate the anti-cancer-immune functions in high- and low-risk patients across the cancer-immune phase, including cancerous antigen release, antigen presentation, T-cell recognition, and cancer cell killing. As shown in Figure 7E, the high-risk group showed significantly stronger immune cell infiltration than the low-risk group (P < 0.05). However, regarding immune cell priming and activation, the high-risk group shown weaker activity than the low-risk group. These results suggest that the promotion of immune cell activation within the high-risk group and the improvement of immune cell penetration within the low-risk group might contribute to better clinical outcomes in patients with OSCC.

### 3.6 Pathway Enrichment Analysis and Tumor Drug Sensitivity Analysis between High- and Low-Risk Groups

**Figure 8:**
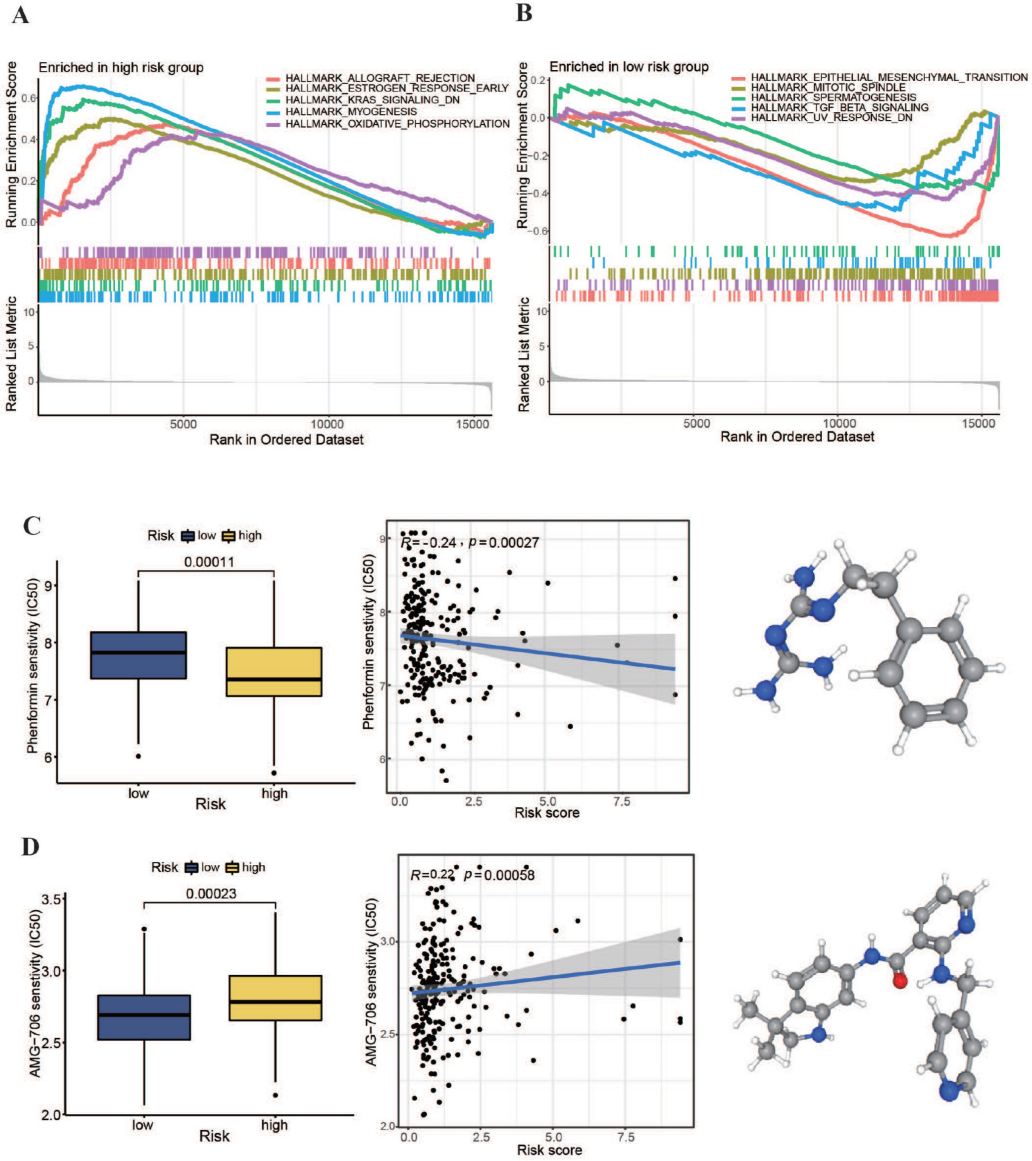
** Pathway enrichment investigation and chemotherapy drug sensitivity analysis within high- and low-risk groups. (A, B)** Pathway enrichment analysis results within high- and low-risk groups. **(C, D)** IC_50_ values of phenformin and AMG-706 for the two risk groups.

We conducted an enrichment study for gene sets to explore potential regulatory processes that differed between high- and low-risk groups. Additionally, we performed a tumor drug sensitivity analysis on TCGA-OSCC patient data to provide a theoretical foundation for personalized treatment. The pathway enrichment results, as illustrated in Figure 8A, revealed significant upregulation of the oxidative phosphorylation pathway in the high-risk group. Furthermore, as depicted in Figure 8C, a comparison of the generally utilized IC_50_ scores of cancer therapeutic drugs between the two categories showed a markedly lower IC_50_ value for the oxidative phosphorylation inhibitor phenformin in the high-risk subgroup (P < 0.001). Moreover, the patient risk scores were significantly negatively associated with phenformin IC_50_ values (r = -0.24, P < 0.001). These findings suggest that phenformin may be an effective therapeutic drug for patients with high-risk OSCC. In the low-risk group, signaling pathways, such as those associated with epithelial-mesenchymal transition and transforming growth factor-beta, were notably upregulated (Figure 8B). Additionally, the comparison revealed that the multikinase inhibitor AMG-706 (motesanib) presented significantly low IC_50_ values in low-risk group (P < 0.001). These results indicate that motesanib might be more effective in patients with low-risk OSCC. As a multikinase inhibitor, motesanib can interfere with various biological functions such as angiogenesis, cell growth, and EMT (Figure 8D). These findings suggest that our m7G methyltransferase-related miRNA prognostic model performs well in guiding drug treatment in patients with OSCC.

### 3.7 Expression Level Detection of m7G Methyltransferase-Related miRNAs in OSCC Tissues

**Figure 9:**
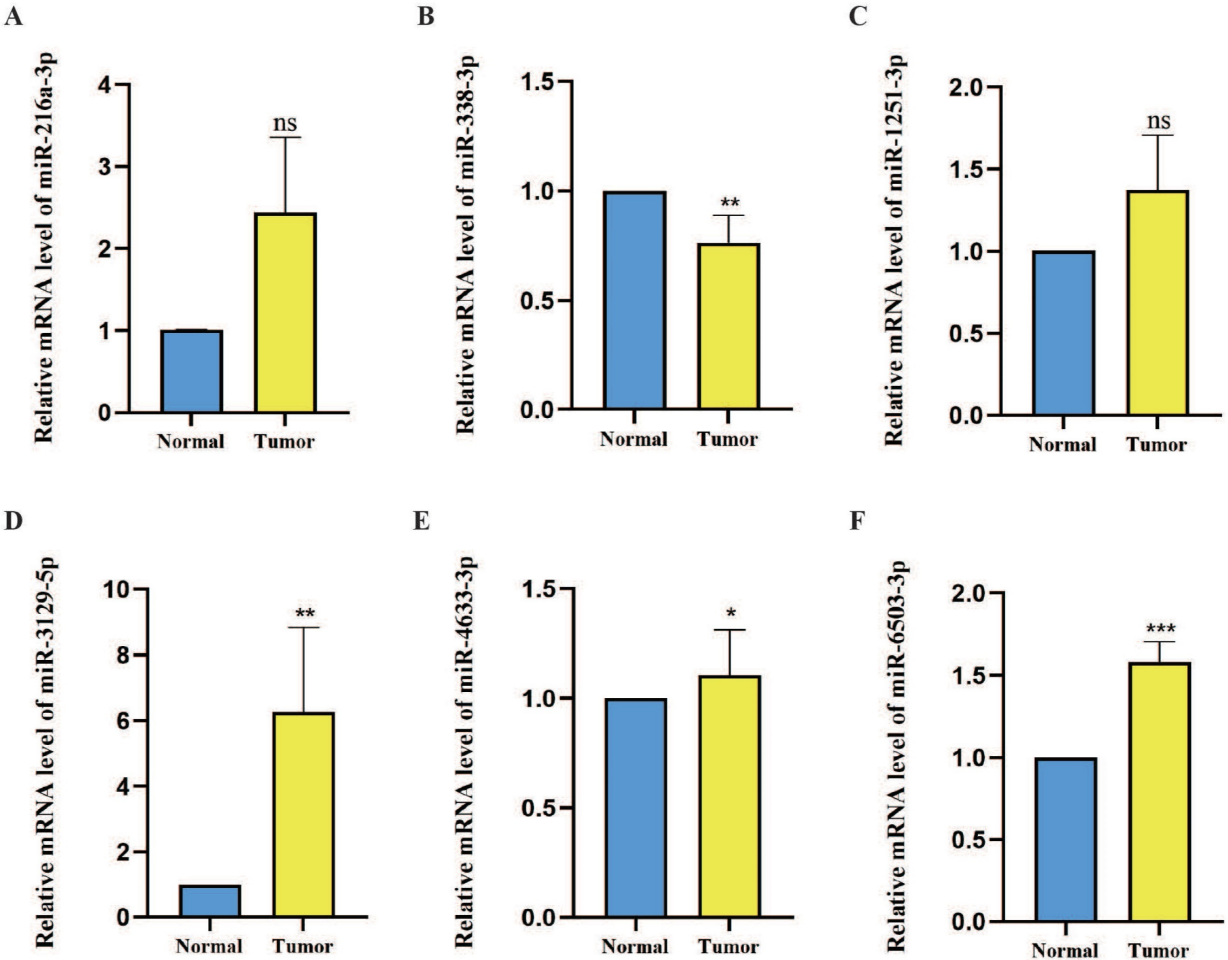
**Expression levels of six candidate miRNAs associated with m7G methyltransferase genes in OSCC tissues. (A-F)** Relative expression levels of six miRNAs were assessed using qPCR within 60 pairs of OSCC tissues and adjacent normal tissues.

The expression levels of six prognostic model m7G methyltransferase-related miRNAs were assessed using qPCR in postoperative OSCC tissues and their corresponding neighboring noncancerous tissues. As shown in Figure 9A-F, the expression levels of hsa-miR-216a-3p, hsa-miR-1251-3p, hsa-miR-3129-5p, hsa-miR-4633-3p, and hsa-miR-6503-3p were elevated in OSCC tissues relative to those in noncancerous tissues. However, hsa-miR-338-3p showed lower expression in OSCC tissues than in noncancerous tissues. However, further statistical analysis revealed that only hsa-miR-338-3p (P < 0.01), hsa-miR-3129-5p (P < 0.01), hsa-miR-4633-3p (P < 0.05), and hsa-miR-6503-3p (P < 0.001) differed significantly between OSCC and noncancerous tissues. Considering the comprehensive analysis of TCGA data, hsa-miR-338-3p and hsa-miR-3129-5p can be considered more reliable miRNAs for the m7G methyltransferase-related miRNA prognosis model genes.

## 4. Discussion

OSCC is a highly heterogeneous tumor. Different tumor progression and prognoses may be observed even in patients with OSCC with similar tumor grading and clinical staging. While there are various treatment methods such as surgery, chemotherapy, and radiotherapy that can, to some extent, improve the survival time of patients with OSCC, the prognosis has been unsatisfactory for those in advanced stages and with metastatic OSCC. The predictive capabilities of some confirmed biomarkers are limited and insufficient for OSCC diagnosis, treatment, and patient survival evaluation. Therefore, exploring new prognostic markers for the accurate prediction of OSCC patient prognosis and providing guidance for personalized treatment to avoid undertreatment and overtreatment are crucial. miRNAs are associated with cancer development and treatment. Studies have suggested a crucial function of m7G methyltransferase-related genes in the incidence and growth of cancer, especially the m7G regulatory factors METTL1 and WDR4. It has been confirmed that the expression of these two factors is significantly upregulated in neck and head squamous carcinoma, leading to an increase in the m7G modification level of tRNA and the promotion of tumor progression 19,30. Subsequently, m7G methyltransferase-associated miRNAs have been increasingly used in cancer research. Models predicting patient prognosis have been successfully constructed using m7G methyltransferase-related miRNAs in liver cancer, breast cancer, renal clear cell carcinoma, and lung adenocarcinoma, confirming their potential value and role as tumor prognostic factors 31-35. However, there have been no reports on m7G methyltransferase-associated miRNAs in OSCC. Therefore, the latent interface of m7G methyltransferase-related miRNAs can be used to determine their probable predictive value.

We used the m7G methyltransferases METTL1 and WDR4 to predict miRNAs associated with m7G methylation. We identified 56 differentially expressed m7G methylation-related miRNAs in OSCC tissues. These miRNAs are primarily involved in the regulation of tumor metabolism and immune-related pathways. Abnormal activation of these signaling pathways induces OSCC growth, invasion, metastasis, and changes in the tumor-resistant microenvironment 36-38. We constructed a novel OSCC risk prognostic model using six representative m7G methylation-related miRNAs (hsa-miR-338-3p, hsa-miR-1251-3p, hsa-miR-4633-3p, hsa-miR-216a-3p, hsa-miR-6503-3p, and hsa-miR-3129-5p). Among these miRNAs, hsa-miR-338-3p, hsa-miR-1251-3p, hsa-miR-4633-3p, hsa-miR-216a-3p, and hsa-miR-6503-3p are considered risk factors for OSCC, whereas hsa-miR-3129-5p is considered a protective factor. Although these six miRNAs have been reported in other cancers, studies on OSCC are limited. Zhao et al. found that miR-338-3p is an oncogene in mucoepidermoid carcinoma 39, while Abbas et al. discovered that miR-216a-3p may promote oral cancer progression by targeting adenylate cyclase 2 (ADCY2) 40. Additionally, Martinez et al. found that the upregulation of miR-6503-3p is associated with decreased stability of primary cilia in thyroid cells, potentially triggering autoimmune thyroid diseases 41. Cao et al. found that the upregulation of lncRNA *MALAT1* mediates doxorubicin immunity and hepatocellular carcinoma progression through miR-3129-5p 42,43. Based on these six m7G methylation-related miRNAs, we constructed a prognostic risk model to develop new strategies for the prognosis and treatment of OSCC. Furthermore, qPCR was used to analyze miRNA expression in OSCC tissues and adjacent normal tissues. The results showed major expression differences in hsa-miR-338-3p, hsa-miR-3129-5p, hsa-miR-4633-3p, and hsa-miR-6503-3p between the OSCC tissues and adjacent normal samples. Through integrated analysis of TCGA dataset results, hsa-miR-338-3p and hsa-miR-3129-5p were found to be the most reliable miRNAs for predicting the m7G methylation-related miRNA model. Specifically, hsa-miR-3129-5p, a protective factor against OSCC, was upregulated in OSCC tissues, possibly inhibiting OSCC progression by enhancing its targeting of WDR4. Conversely, hsa-miR-338-3p, a risk factor for OSCC, was significantly downregulated in OSCC tissues, consistent with a previous analysis.

Owing to the high heterogeneity and complex etiological factors of OSCC tumors, TNM staging is also challenging for predicting or describing the individual risks and prognoses of patients with OSCC at the same stage. In comparison with TNM staging, our constructed m7G methyltransferase-related miRNA prognostic model exhibited self-assessment values for 1, 3, and 5 years prognosis, all exceeding 0.7. This indicated that the prognostic model built on m7G methyltransferase-related miRNAs exhibited excellent presentation for predicting the diagnosis of patients with OSCC. Furthermore, the concordance index curve demonstrated that our prognostic model was more reliable for assessing patient prognosis than TNM staging and other clinical indicators. Independent prognostic analysis confirmed the effectiveness of the diagnostic model as a self-regulating indicator for patients with OSCC.

The possible regulatory processes underlying the differences between the two subgroups were investigated. Notably, we found an upregulated oxidative phosphorylation pathway in the high-risk group compared with the low-risk group. This suggests that inhibitors of oxidative phosphorylation might have had a stronger antitumor effect in the high-risk group of patients with OSCC. Additionally, higher enrichment of gene sets associated with Parkinson's disease and myocardial contraction was found in this group, suggesting that patients with these conditions may have higher risk scores. Interestingly, the potential associations between these diseases open more possibilities for the development of new drugs for the treatment of OSCC. For instance, Eizuka et al. discovered that L-3,4-dihydroxyphenylalanine, a permitted drug for the treatment of Parkinson's disease, could potentially become a new drug for the treatment of OSCC. This is because it hinders OSCC development by down-regulating SYT12 expression 44,45.

The tumor microenvironment varies among patients, and these differences contribute to the proliferation, migration, and invasion capabilities of OSCC cells, explaining the significant variations in prognosis among patients 46-48. High-risk patients exhibited increased immune penetration, indicating higher immunity. However, considering patient survival curves, this abnormally active immune status did not improve patient prognosis. Additionally, most immune-related gene expression levels were higher in the high-risk group, suggesting that increased inflammation and elevated immune checkpoint activity may contribute to immunodeficiency, partially explaining the poor prognosis of high-risk patients with OSCC. Moreover, compared to low-risk patients, high-risk patients had relatively weaker immune cell activation functions, although immune permeation into the tumor was significantly stronger. This implies that promoting immune cell activation in high-risk patients and improving immune cell penetration in low-risk patients may improve the clinical prognosis of patients with OSCC 49. Furthermore, we propose that the upregulation of immune checkpoint-associated genes in high-risk patients may represent potential targets for immunotherapy in this population. Notably, TNFRSF14 (HVEM) and its receptor BTLA (CD272) were significantly upregulated in high-risk patients. The upregulation of TNFRSF14 in tumor cells contributes to the inhibition of antitumor resistance by BTLA, leading to disease development and worse diagnostic outcomes 50,51. Therefore, targeting BTLA-HVEM in high-risk patients may be a feasible immunotherapeutic approach.

Currently, radiotherapy and chemotherapy are the main approaches for the postoperative treatment of patients with OSCC. In chemotherapy, platinum-based drugs such as 5-fluorouracil, paclitaxel, and doxorubicin are the most commonly used drugs. However, owing to individual differences among patients and multidrug resistance, the application of these drugs in OSCC treatment is limited, often leading to chemotherapy failure 52,53. Therefore, to guide personalized treatment more effectively, we compared the IC_50_ values of general anticancer drugs between the two subgroups. These results indicated that phenformin may be more suitable for the treatment of high-risk patients. Phenformin, an oxidative phosphorylation inhibitor, disrupts the energy metabolism of tumor cells and inhibits angiogenesis within tumors by inhibiting oxidative phosphorylation, effectively suppressing OSCC growth 35,53-55. Conversely, motesanib (AMG-706) may be more suitable for treating low-risk patient subgroups. It is a potent multi-kinase inhibitor that can overcome resistance to doxorubicin and paclitaxel by inhibiting ABCB1 efflux action 43,56-58. Therefore, motesanib is expected to serve as an adjuvant chemotherapy drug, enhancing the sensitivity of low-risk patients to chemotherapeutic drugs and increasing the effectiveness of treatment.

This study has a few limitations. Firstly, the analyzed data were derived from public databases with limited sample sizes. In addition, experimental data were lacking. Therefore, future studies should explore the functions and processes of m7G methyltransferase-associated miRNAs in patients with OSCC. Future studies should also focus on elucidating the deep-seated mechanisms by which m7G methyltransferase-related miRNAs trigger the activation of immune checkpoint molecules to uncover potential targets for specific immunotherapy in OSCC.

In conclusion, our results revealed the diagnostic score of m7G methyltransferase-related miRNAs. We also developed an m7G methyltransferase-related miRNA prognostic model associated with distinct clinical outcomes and immune characteristics in patients with OSCC. Our findings lay the foundation for developing new prognostic models for OSCC, with important clinical significance for personalized treatment strategies in patients with OSCC.

## Supplementary Material

Supplementary table.

## Figures and Tables

**Table 1:** The six candidate miRNAs and their corresponding coefficients involved in the model construction.

miRNAs	Coefficient
hsa-miR-338-3p	0.326343
hsa-miR-1251-3p	1.59744
hsa-miR-4633-3p	2.288034
hsa-miR-216a-3p	0.742978
hsa-miR-6503-3p	0.347863
hsa-miR-3129-5p	-0.49423
