# Supplementary material for: Establishment and assessment of an oral squamous cell carcinoma N7-methylguanosine methyltransferase associated microRNA prognostic model
Source: J Cancer. 2024 Sep 30;15(18):6022–37. doi: 10.7150/jca.98350 (PMC11493003; doi:10.7150/jca.98350)
Supplement: Supplementary file 1 — Supplementary table. [file jcav15p6022s1.pdf]

Supplementary Table 1: Details of the primer sequences used in this research

Primer	Sequences
hsa-miR-216a-3p	Forward: 5'-ACACTCCAGCTGGGTCACAGTGGTCTCTGGGA-3'<br>RT: 5'-CTCAACTGGTGTCGTGGAGTCGGCAATTCAGTTGAGATAATCC-3'
hsa-miR-338-3p	Forward: 5'-ACACTCCAGCTGGGTCCAGCATCAGTGATTTT-3'<br>RT: 5'-CTCAACTGGTGTCGTGGAGTCGGCAATTCAGTTGAGCAACAAA-3'
hsa-miR-1251-3p	Forward: 5'-ACACTCCAGCTGGGCGCTTTGCTCAGCCAGT-3'<br>RT: 5'-CTCAACTGGTGTCGTGGAGTCGGCAATTCAGTTGAGCTACACT-3'
hsa-miR-3129-5p	Forward: 5'-ACACTCCAGCTGGGGCAGTAGTGTAGAGATTG-3'<br>RT: 5'-CTCAACTGGTGTCGTGGAGTCGGCAATTCAGTTGAGAAACCAA-3'
hsa-miR-4633-3p	Forward: 5'-ACACTCCAGCTGGGAGGAGCTAGCCAGGCATA-3'<br>RT: 5'-CTCAACTGGTGTCGTGGAGTCGGCAATTCAGTTGAGTGCATAT-3'
hsa-miR-6503-3p	Forward: 5'-ACACTCCAGCTGGGGGGACTAGGATGCAGAC-3'<br>RT: 5'-CTCAACTGGTGTCGTGGAGTCGGCAATTCAGTTGAGGGAGGTC-3'
Universal Reverse	5'-TGTCGTGGAGTCGGCAATTC-3'
U6	Forward: 5'-GCTTCGGCAGCACATATACTAAAAT-3'<br>Reverse: 5'-CGCTTCACGAATTTGCGTGTCAT-3'<br>RT: 5'-CGCTTCACGAATTTGCGTGTCAT-3'
